# Combination therapy for melanoma with BRAF/MEK inhibitor and immune checkpoint inhibitor: a mathematical model

**DOI:** 10.1186/s12918-017-0446-9

**Published:** 2017-07-19

**Authors:** Xiulan Lai, Avner Friedman

**Affiliations:** 1 Institute for Mathematical Sciences, Renmin University of China, Beijing, 100872 People’s Republic of China; 2 Mathematical Bioscience Institute & Department of Mathematics, Ohio State University, Columbus, 43210 OH USA

**Keywords:** Melanoma, Mathematical modeling, BRAF/MEK inhibitor, PD-1 inhibitor, Combination therapy

## Abstract

**Background:**

The B-raf gene is mutated in up to 66% of human malignant melanomas, and its protein product, BRAF kinase, is a key part of RAS-RAF-MEK-ERK (MAPK) pathway of cancer cell proliferation. BRAF-targeted therapy induces significant responses in the majority of patients, and the combination BRAF/MEK inhibitor enhances clinical efficacy, but the response to BRAF inhibitor and to BRAF/MEK inhibitor is short lived. On the other hand, treatment of melanoma with an immune checkpoint inhibitor, such as anti-PD-1, has lower response rate but the response is much more durable, lasting for years. For this reason, it was suggested that combination of BRAF/MEK and PD-1 inhibitors will significantly improve overall survival time.

**Results:**

This paper develops a mathematical model to address the question of the correlation between BRAF/MEK inhibitor and PD-1 inhibitor in melanoma therapy. The model includes dendritic and cancer cells, CD 4^+^ and CD 8^+^ T cells, MDSC cells, interleukins IL-12, IL-2, IL-6, IL-10 and TGF- *β*, PD-1 and PD-L1, and the two drugs: BRAF/MEK inhibitor (with concentration *γ*_*B*_) and PD-1 inhibitor (with concentration *γ*_*A*_). The model is represented by a system of partial differential equations, and is used to develop an efficacy map for the combined concentrations (*γ*_*B*_,*γ*_*A*_). It is shown that the two drugs are positively correlated if *γ*_*B*_ and *γ*_*A*_ are at low doses, that is, the growth of the tumor volume decreases if either *γ*_*B*_ or *γ*_*A*_ is increased. On the other hand, the two drugs are antagonistic at some high doses, that is, there are zones of (*γ*_*B*_,*γ*_*A*_) where an increase in one of the two drugs will increase the tumor volume growth, rather than decrease it.

**Conclusions:**

It will be important to identify, by animal experiments or by early clinical trials, the zones of (*γ*_*B*_,*γ*_*A*_) where antagonism occurs, in order to avoid these zones in more advanced clinical trials.

## Background

PD-1 is an immunoinhibitory receptor predominantly expressed on activated T cells [1, 2]. Its ligand PD-L1 is upregulated on the same activated T cells, and is also expressed by myeloid-derived suppressor cells (MDSCs) [2–5] and in some human cancer cells, including melanoma, lung cancer, colon cancer, and leukemia [2, 3]. The complex PD-1-PD-L1 is known to inhibit T cell function [1]. Immune checkpoints are regulatory pathways in the immune system that inhibit its active response against specific targets. In the case of cancer, the complex PD-1-PD-L1 functions as an immune checkpoint for anti-tumor T cells. There has been much progress in recent years in developing checkpoint inhibitors, primarily anti-PD-1 and anti-PD-L1 [6].

The B-raf gene is mutated in up to 66% of human malignant melanomas, and its protein product, BRAF kinase, is a key part of the RAS-RAF-MEK-ERK (MAPK) pathway of cancer cell proliferation [7]. BRAF-targeted therapy induces significant response in the majority of patients but the response is short lived (about 6 months) [7–9]. Initial clinical trials indicate that concurrent inhibition of BRAF with MEK decreases MAPK-driven acquired resistance, resulting in enhanced clinical efficacy and decreased toxicity [10, 11]. This provides a rational for using combined BRAF/MEK inhibition instead of BRAF inhibition alone [11]. Treatment of melanoma with immune checkpoint inhibitors has a lower response rate compared to treatment with BRAF/MEK inhibitors, but the response tends to be more durable, lasting for years [11–13]. It was therefore suggested that BRAF/MEK-targeted therapy may synergize with the PD-1 pathway blockade to enhance anti-tumor immunity [4, 11, 14, 15]. Meta-Analysis of randomized clinical trials show that compared with other treatments of advanced BRAF-mutated melanoma, combined BRAF/MEK and PD-1 inhibitions significantly improved overall survival time [16].

In this paper we develop a mathematical model to address the efficacy of the combination of BRAF/MEK inhibitor (BRAF/MEKi) and anti-PD-1 (e.g. nivolumab). The model includes several types of T cells, MDSCs, and dendritic cells, as well as signaling molecules involved in the crosstalks among these cells.

Melanoma-derived factors alter the maturation and activation of tissue-resident dendritic cells, thus favoring tumor immune escape [17]. In BRAF mutant melanoma, BRAF inhibitor restores the compromised dendritic cells function, and, in particular, the production of IL-12 by dendritic cells [18]. Although MEK inhibitor (e.g. trametinib), as single agent, negatively impacts DC function, when combined with BRAF inhibitor (e.g. vemurafenib or dabrafenib), the functionality of DCs is restored, as well as their production of IL-12 [18, 19].

Dendritic cell-derived IL-12 activates effector T cells (Th1 and CD 8^+^ T cells) [20, 21]. Th1 produces IL-2 which further promotes proliferation of effector T cells. CD 4^+^ T cells (Th1) can kill cancer cell directly, for example, through FAS- or TRAIL-dependent pathway [22–25], while CD 8^+^ T cell is more effective in killing cancer cells [26]. Cancer cells suppress the functions of effector T cells by producing immunosuppressor cytokines TGF- *β*, IL-6, CCL2 and IL-10 [27]. IL-10 inhibits the activation of Th1 and CD 8^+^ T cells [27]. IL-6 and CCL2 recruit MDSCs into tumor [19, 28, 29], and MDSCs produce TGF- *β* and IL-10. IL-6 and CCL2 also recruit T regulatory T cells (Tregs) [15, 28, 29]. TGF- *β* is produced not only by cancer cells and MDSCs, but also by Tregs [30], and Tregs become activated by TGF- *β* [30, 31]. Tregs modulate Th1 and CD 8^+^ T cells [30], thus promoting tumor growth.

One of the checkpoints on T cells is the membrane protein PD-1. Its ligand PD-L1 is expressed on activated effector T cells, on MDSCs and on cancer cells [2–5]. The complex PD-1-PD-L1 inhibits the function of effector T cells [1], but enhances the activation of Tregs [32] and thus promoting cancer.

**Fig. 1:**
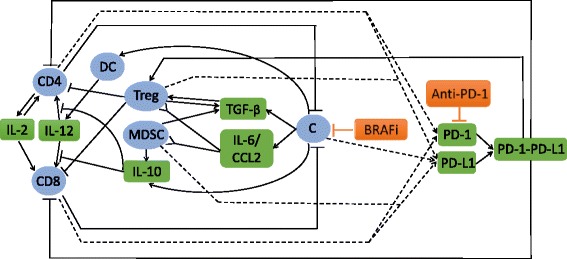
Interaction of immune cells with cancer cells. *Sharp arrows* indicate proliferation/activation, blocked arrows indicate killing/blocking, *inverted sharp arrows* indicate recruitment/chemoattraction, and *dashed lines* indicate proteins on T cells, MDSCs and cancer cells

The above interactions between cancer cells and the immune cells are summarized in Fig. 1. The mathematical model developed in the present paper is based on Fig. 1, and it includes BRAF/MEK and PD-1 inhibitors. Simulations of the model show that at low doses the two drugs are positively correlated, in the sense that the tumor volume decreases as each of the drugs is increased. However, at high doses the two drugs may become antagonistic, that is, an increase in dose of one of the drugs may actually result in an increase in the tumor volume.

## Methods

### Mathematical model

The mathematical model is based on the network shown in Fig. 1. The list of variables is given in Table 1. Since CCL2 and IL-6 are both produced by cancer cells and both recruit MDSCs and Tregs into tumor environment, we shall consider, for simplicity, only IL-6 in our model.

**Table 1:** List of variables (in units of g/ cm^3^)

Notation	Description
*H*	HMGB-1 concentration
*N*_*C*_	Density of necrotic cancer cells
*D*	Density of DCs
*T*_1_	Density of activated CD 4^+^ T cells
*T*_8_	Density of activated CD 8^+^ T cells
*T*_*r*_	Density of activated Treg cells
*M*	Density of activated MDSCs
*C*	Density of cancer cells
*I*_12_	IL-12 concentration
*I*_2_	IL-2 concentration
*T*_*β*_	TGF- *β* concentration
*I*_6_	IL-6 concentration
*I*_10_	IL-10 concentration
*P*	PD-1 concentration
*L*	PD-L1 concentration
*Q*	PD-1-PD-L1 concentration
*A*	Anti-PD-1 concentration
*B*	BRAF/MEK inhibitor concentration

We assume that the total density of cells within the tumor remains constant in space and time:

1 $$  C+D+T_{1}+T_{8}+T_{r}+M=\text{constant}.  $$

We assume that the density of debris of dead cells from necrosis or apoptosis is constant. We also assume that the densities of immature dendritic cells and naive CD 4^+^ and CD 8^+^ T cells remain constant throughout the tumor tissue. Under the assumption (1), proliferation of cancer cells and immigration of immune cells into the tumor, give rise to internal pressure which results in cells movement. We assume that all the cells move with the same velocity, **u**; **u** depends on space and time and will be taken in unit of cm/day. We also assume that all the cells undergo dispersion (i.e., diffusion), and that all the cytokines and anti-tumor drugs are diffusing within the tumor.

#### Equation for DCs (*D*)

By necrotic cancer cells (*N*_*C*_) we mean cancer cells undergoing the process of necrosis. Necrotic cancer cells release HMGB-1 (*H*) [33]. We model the dynamics of *N*_*C*_ and *H* by the following equations:

$$ {{}\begin{aligned} &\frac{\partial N_{C}}{\partial t}+\underbrace{\nabla \cdot (\mathbf{u} N_{C})}_{\text{velocity}}-\underbrace{\delta_{N_{C}}\nabla^{2} N_{C}}_{\text{diffusion}}=\underbrace{\lambda_{N_{C}C}C}_{\textrm{derived from alife cancer cells}}\!-d_{N_{C}}N_{C},\\ &\frac{\partial H}{\partial t}-\underbrace{\delta_{H}\nabla^{2} H}_{\text{diffusion}}=\underbrace{\lambda_{HN_{C}}N_{C}}_{\textrm{released from necrotic cancer cells}}-\underbrace{d_{H}H}_{\text{degradation}}, \end{aligned}}  $$

where $\lambda _{N_{C}C}$ is the rate at which cancer cells become necrotic, *d*_*N*_ is the rate at which necrotic cells turn into debris, and $\lambda _{HN_{C}}$ is the rate at which necrotic cells produce HMGB-1. We note that although molecules like HMGB-1, or other proteins, may be affected by the velocity **u**, their diffusion coefficients are several order of magnitude larger than the diffusion coefficients of cells, hence their velocity term may be neglected. The degradation of HMGB-1 is fast (∼0.01/day) [34], and we assume that the process of necrosis is also fast. We may then approximate the two dynamical equations by the steady staten$\lambda _{N_{C}C}C-d_{N_{C}}N_{C}=0$ and $\lambda _{HN_{C}}N_{C}-d_{H}H=0$, so that *H* is proportional to *C*.

Dendritic cells are activated by HMGB-1 [35, 36]. Hence, the activation rate of immature dendritic cells, with density *D*_0_, is proportional to $D_{0}\frac {H}{K_{H}+H}$, or to $D_{0}\frac {C}{K_{C}+C}$, since *H* is proportional to *C*. Here, the Michaelis-Menten law is used to account for the limited rate of receptor recycling time which takes place in the process of DCs activation. Hence, the dynamics of DCs is given by

2 $$\begin{array}{*{20}l} \frac{\partial D}{\partial t}+\underbrace{\nabla \cdot (\mathbf{u} D)}_{\text{velocity}}-\underbrace{\delta_{D}\nabla^{2} D}_{\text{diffusion}}=\underbrace{\lambda_{DC}D_{0}\frac{C}{K_{C}+C}}_{\textrm{activation by HMGB-1}}-\underbrace{d_{D}D}_{\text{death}}, \end{array} $$

where *δ*_*D*_ is the diffusion coefficient and *d*_*D*_ is the death rate of DCs.

#### Equation for CD 4^+^ T cells (*T*_1_)

Naive CD 4^+^ T cells differentiate into Th1 cells (*T*_1_) under IL-12 (*I*_12_) environment [20, 21], while IL-10 and Tregs inhibit the differentiation of naive CD 4^+^ T cells into *T*_1_ cells [27, 30]. The proliferation of activated *T*_1_ cells is enhanced by IL-2. Both processes of activation and proliferation of *T*_1_ are assumed to be inhibited by PD-1-PD-L1 (*Q*) by a factor $\frac {1}{1+Q/K_{TQ}}$. Hence *T*_1_ satisfies the following equation:

3 $$ {{}\begin{aligned} \frac{\partial T_{1}}{\partial t}\! +\!\nabla \!\cdot\! (\mathbf{u} T_{1})\,-\,\delta_{T}\nabla^{2} T_{1}&= \underbrace{\left(\lambda_{T_{1}I_{12}}T_{10}\cdot \frac{I_{12}}{K_{I_{12}}+I_{12}}\right.}_{\textrm{activation by IL-12}}\cdot \underbrace{\frac{1}{1+I_{10}/K_{TI_{10}}}}_{\textrm{inhibition by IL-10}}\\ &\quad\times \underbrace{\frac{1}{1+T_{r}/K_{TT_{r}}}}_{\textrm{inhibition by Tregs}} \\ &\quad+\!\!\!\underbrace{\left. \lambda_{T_{1}I_{2}}T_{1}\!\frac{I_{2}}{K_{I_{2}}+I_{2}}\right)}_{\textrm{IL-2-induced proliferation}}\times \!\!\!\!\! \underbrace{\frac{1}{1+Q/K_{TQ}}}_{\textrm{inhibition by PD-1-PD-L1}}\\ &\quad-\underbrace{d_{T_{1}}T_{1}}_{\text{death}}, \end{aligned}}  $$

where *T*_10_ is the density of the naive CD 4^+^ T cells.

#### Equation for activated CD 8^+^ T cells (*T*_8_)

Inactive CD 8^+^ T cells are activated by IL-12 [20, 21], and this process is resisted by IL-10 and Tregs [27, 30]. IL-2 enhances the proliferation of activated CD 8^+^ T cells. Similarly to the equation for *T*_1_, *T*_8_ satisfies the following equation:

4 $$ {{}\begin{aligned} \frac{\partial T_{8}}{\partial t}+\nabla \cdot (\mathbf{u} T_{8})\,-\,\delta_{T}\nabla^{2} T_{8}&=\underbrace{\left(\lambda_{T_{8}I_{12}}T_{80}\cdot \frac{I_{12}}{K_{I_{12}}+I_{12}}\right.}_{\text{activation by IL-12}}\\ &\quad\times \underbrace{\frac{1}{1+I_{10}/K_{TI_{10}}}}_{\text{inhibition by IL-10}}\cdot \!\underbrace{\frac{1}{1+T_{r}/K_{TT_{r}}}}_{\text{inhibition by Tregs}}\\ &\quad+\underbrace{\left.\lambda_{T_{8}I_{2}}T_{8}\frac{I_{2}}{K_{I_{2}}+I_{2}}\right)}_{\text{IL-2-induced proliferation}}\\ &\quad \times \underbrace{\frac{1}{1+Q/K_{TQ}}}_{\text{inhibition by PD-1-PD-L1}}-\underbrace{d_{T_{8}}T_{8}}_{\text{death}}, \end{aligned}}  $$

where *T*_80_ is the density of the inactive CD 8^+^ T cells.

#### Equation for activated Tregs (*T*_*r*_)

Naive CD 4^+^ T cells differentiate into Tregs (*T*_*r*_) under activation by Fox3+ transcription factor. The complex PD-1-PD-L1 enhances the expression of PTEN, which results in upregulation of Fox3+, and hence in increased production of Tregs [32]. The production of *T*_*r*_ is also enhanced by TGF- *β* (*T*_*β*_) [30, 31]. The activated Tregs are recruited into tumor by tumor-derived immunosuppressive cytokine IL-6 (and CCL2)[15, 28, 29]. Representing this chemoattraction by ∇·(*χ* *T*_*r*_∇*I*_6_), we get the following equation for *T*_*r*_:

5 $$ {{}\begin{aligned} \frac{\partial T_{r}}{\partial t}+\nabla \cdot (\mathbf{u} T_{r})-\delta_{T}\nabla^{2} T_{r}=&T_{10}\!\!\!\!\underbrace{\left(\lambda_{T_{r}T_{\beta}}\frac{T_{\beta}}{K_{T_{\beta}}+T_{\beta}}\right.}_{\text{TGF-\(\upbeta\)-induced proliferation}}\!\,+\,\!\underbrace{\lambda_{T_{r}Q}\left.\frac{Q}{K_{Q}+Q}\right)}_{\text{promotion by PD-1-PD-L1}}\\ &-\underbrace{\nabla \cdot (\chi T_{r}\nabla I_{6})}_{\text{recruited by IL-6}}-\underbrace{d_{T_{r}}T_{r}}_{\text{death}}. \end{aligned}}  $$

#### Equation for activated MDSCs (*M*)

Tumor recruits macrophages and “educates” them to become tumor-associated-macrophages (TAMs), which behave like MDSCs [37, 38]. MDSCs are also chemotactically attracted to the tumor microenvironment by IL-6 (and CCL2) [15, 19, 28, 29, 39]. As in [40], the Eq. of MDSCs is taken to be the following form:

6 $$ \begin{aligned} \frac{\partial M}{\partial t}+\nabla \cdot (\mathbf{u} M)-\delta_{M}\nabla^{2} M=\lambda_{M}(M_{0}-M)\\\times\frac{I_{6}}{K_{I_{6}}+I_{6}}-\underbrace{\nabla \cdot (\chi M\nabla I_{6})}_{\text{recruited by IL-6}}-\underbrace{d_{M}M}_{\text{death}}, \end{aligned}  $$

where *M*_0_ is the source/influx of macrophages from the blood.

#### Equation for tumor cells (*C*)

Cancer cells are killed by *T*_1_ and *T*_8_ cells. We assume a logistic growth with carrying capacity (*C*_*M*_) in order to account for competition for space among cancer cells. BRAF/MEK inhibitor (*B*), for example vemurafenib/dabrafenib, is used for treatment of advanced melanoma. Its mechanism of action involves selective inhibition of the mutated BRAF kinase that leads to reduced signaling through the aberrant RAS-RAF-MEK-ERK (MAPK) pathway. We assume that BRAF/MEK inhibitor suppresses the abnormal proliferation of tumor cells by a factor $\frac {1}{1+B/K_{CB}}$. Then, the equation for *C* takes the form:

7 $$ {{}\begin{aligned} \frac{\partial C}{\partial t}+\nabla \cdot (\mathbf{u} C)-\delta_{C}\nabla^{2} C=&\underbrace{\lambda_{C}C\left(1-\frac{C}{C_{M}}\right)}_{\text{proliferation}}\cdot\!\!\!\!\!\!\!\underbrace{\frac{1}{1+B/K_{CB}}}_{\text{inhibition by BRAF/MEKi}}\\ &-\underbrace{(\eta_{1} T_{1}C+\eta_{8} T_{8}C)}_{\text{killing by T cells}}-\underbrace{d_{C}C}_{\text{death}}, \end{aligned}}  $$

where *η*_1_ and *η*_8_ are the killing rates of cancer cells by *T*_1_ and *T*_8_, and *d*_*C*_ is the natural death rate of cancer cells.

#### Equation for IL-12 (*I*_12_)

The proinflammatory cytokine IL-12 is secreted by activated DCs [20, 21], so that

$$\begin{array}{@{}rcl@{}}  \frac{\partial I_{12}}{\partial t}-\delta_{I_{12}}\nabla^{2} I_{12}&=&\underbrace{\lambda_{I_{12}D}D}_{\text{production by DCs}}-\underbrace{d_{I_{12}}I_{12}}_{\text{degradation}}. \end{array} $$

The maturation and activation of dendritic cells is interrupted by melanoma cells, which means that the production rate coefficient $\lambda _{I_{12}D}$ is small. However, in BRAF mutant melanoma, BRAF inhibitor alone or in combination with MEK inhibitor, restores the compromised dendritic cells function, and in particular, the production of IL-12 by dendritic cells [18, 19], and the corresponding equation for *I*_12_ then takes the form:

8 $$\begin{array}{@{}rcl@{}}  \frac{\partial I_{12}}{\partial t}\!-\delta_{I_{12}}\nabla^{2} I_{12}&\,=\,&\underbrace{\lambda_{I_{12}D}D\cdot \left(\!1\,+\, \lambda_{I_{12}B}\frac{B}{K_{B}+B}\!\right)}_{\text{production by DCs}}-\!\underbrace{d_{I_{12}}I_{12}}_{\text{degradation}}.\\ \end{array} $$

#### Equation for IL-2 (*I*_2_)

IL-2 is produced by activated CD 4^+^ T cells (*T*_1_) [21]. Hence,

9 $$\begin{array}{@{}rcl@{}}  \frac{\partial I_{2}}{\partial t}-\delta_{I_{2}}\nabla^{2} I_{2}&=&\underbrace{\lambda_{I_{2}T_{1}}T_{1}}_{\text{production by \(T_{1}\)}}-\underbrace{d_{I_{2}}I_{2}}_{\text{degradation}}. \end{array} $$

#### Equation for TGF- *β* (*T*_*β*_)

TGF- *β* is produced by tumor cells [27], MDSCs [31, 41, 42] and Tregs [30]:

10 $$ {}\begin{aligned} \frac{\partial T_{\beta}}{\partial t}-\delta_{T_{\beta}}\nabla^{2} T_{\beta}=&\underbrace{\lambda_{T_{\beta}C}C}_{\text{production by cancer cells}}+\underbrace{\lambda_{T_{\beta}T_{r}}T_{r}}_{\text{production by Tregs}}\\ &+\underbrace{\lambda_{T_{\beta}M}M}_{\text{production by MDSCs}}-\underbrace{d_{T_{\beta}}T_{\beta}}_{\text{degradation}}. \end{aligned}  $$

#### Equation for IL-6 (*I*_6_)

IL-6 is produced by cancer cells [15, 19, 28], so that

11 $$\begin{array}{@{}rcl@{}}  \frac{\partial I_{6}}{\partial t}-\delta_{I_{6}}\nabla^{2} I_{6}&=&\underbrace{\lambda_{I_{6}C}C}_{\text{production by cancer cells}} -\underbrace{d_{I_{6}}I_{6}}_{\text{degradation}}. \end{array} $$

#### Equation for IL-10 (*I*_10_)

IL-10 is produced by cancer cells and MDSCs [27]. Hence it satisfies the following equation:

12 $$ {}\begin{aligned} \frac{\partial I_{10}}{\partial t}-\delta_{I_{10}}\nabla^{2} I_{10}=&\!\!\!\underbrace{\lambda_{I_{10}C}C}_{\text{production by cancer cells}} +\underbrace{\lambda_{I_{10}M}M}_{\text{production by MDSCs}}\\ &-\underbrace{d_{I_{10}}I_{10}}_{\text{degradation}}. \end{aligned}  $$

#### Equation for PD-1 (*P*), PD-L1 (*L*) and PD-1-PD-L1 (*Q*)

PD-1 is expressed on the surface of activated CD 4^+^ T cells, activated CD 8^+^ T cells and Tregs. We assume that the number of PD-1 per cell is the same for *T*_1_ and *T*_8_ cells, but is smaller, by a factor *ε*_*T*_, for *T*_*r*_ cells. If we denote by *ρ*_*P*_ the ratio between the mass of one PD-1 protein to the mass of one T cell, then

$$\begin{array}{@{}rcl@{}} P=\rho_{P}(T_{1}+T_{8}+\varepsilon_{T} T_{r}). \end{array} $$

The coefficient *ρ*_*P*_ is constant when no anti-PD-1 drug is administered. And in this case, to a change in *T*=*T*_1_+*T*_8_+*ε*_*T*_ *T*_*r*_, given by $\frac {\partial T}{\partial t}$, there corresponds a change of *P*, given by $\rho _{P}\frac {\partial T}{\partial t}$. For the same reason, ∇·(**u** *P*)=*ρ*_*P*_∇·(**u** *T*) and ∇^2^ *P*=*ρ*_*P*_∇^2^ *T* when no anti-PD-1 drug is injected. Hence, *P* satisfies the equation

$$\begin{array}{*{20}l} {}\frac{\partial P}{\partial t}+\nabla \cdot (\mathbf{u} P)-\delta_{T} \nabla^{2} P=&\rho_{P}\left[\frac{\partial(T_{1}+T_{8}+\varepsilon_{T}T_{r})}{\partial t} +\nabla \right.\\& \times (\mathbf{u} (T_{1}+T_{8}+\varepsilon_{T}T_{r})) &\\&\left.-\delta_{T}\nabla^{2} (T_{1}+T_{8}+\varepsilon_{T}T_{r})\right]. \end{array} $$

Recalling Eqs. (3)-(5) for *T*_1_,*T*_8_ and *T*_*r*_, we get

$$ {{}\begin{aligned} \frac{\partial P}{\partial t}+&\nabla \cdot (\mathbf{u} P)-\delta_{T} \nabla^{2} P\\ =&\rho_{P}\left[(\lambda_{T_{1}I_{12}}T_{10}+\lambda_{T_{8}I_{12}}T_{80})\frac{I_{12}}{K_{I_{12}}+I_{12}}\cdot \frac{1}{1+I_{10}/K_{TI_{10}}} \right.\\ & \times \frac{1}{1+T_{r}/K_{TT_{r}}}\,+\,\left. (\lambda_{T_{1}I_{2}}T_{1}\,+\,\lambda_{T_{8}I_{2}}T_{8})\frac{I_{2}}{K_{I_{2}}+I_{2}}\!\right] \!\times \frac{1}{1+Q/K_{TQ}}\\ &+\rho_{P}\varepsilon_{T}T_{10}\cdot\left(\lambda_{T_{r}T_{\beta}}\frac{T_{\beta}}{K_{T_{\beta}}+T_{\beta}}+\lambda_{T_{r}Q}\frac{Q}{K_{Q}+Q}\right)\\ &-\rho_{P}\left[(d_{T_{1}}T_{1}+d_{T_{8}}T_{8}+\varepsilon_{T}d_{T_{r}}T_{r})+\varepsilon_{T}\delta_{TI_{6}}\nabla \cdot (T_{r}\nabla I_{6})\right] \end{aligned}}  $$

When anti-PD-1 drug (*A*) is applied, PD-1 is depleted (or blocked) by *A*. In this case, the ratio $\frac {P}{T_{1}+T_{8}+\varepsilon _{T}T_{r}}$ may change. In order to include in the model both cases of with and without anti-PD-1, we replace *ρ*_*P*_ in the previous equation by $\frac {P}{T_{1}+T_{8}+\varepsilon _{T}T_{r}}$. Hence,

13 $$ {{}\begin{aligned} \frac{\partial P}{\partial t}+&\nabla \cdot (\mathbf{u} P)-\delta_{T} \nabla^{2} P\\ =&\frac{P}{T_{1}+T_{8}+\varepsilon_{T}T_{r}}\!\left[\!(\lambda_{T_{1}I_{12}}T_{10}\,+\,\lambda_{T_{8}I_{12}}T_{80})\frac{I_{12}}{K_{I_{12}}+I_{12}}\cdot \frac{1}{1+I_{10}/K_{TI_{10}}} \right.\\ & \times \frac{1}{1+T_{r}/K_{TT_{r}}}+\left. (\lambda_{T_{1}I_{2}}T_{1}+\lambda_{T_{8}I_{2}}T_{8})\frac{I_{2}}{K_{I_{2}}+I_{2}}\right] \times \frac{1}{1+Q/K_{TQ}}\\ &+\frac{P}{T_{1}+T_{8}+\varepsilon_{T}T_{r}}\varepsilon_{T}T_{10}\cdot\left(\lambda_{T_{r}T_{\beta}}\frac{T_{\beta}}{K_{T_{\beta}}+T_{\beta}}+\lambda_{T_{r}Q}\frac{Q}{K_{Q}+Q}\right)\\ &-\frac{P}{T_{1}\!+T_{8}\,+\,\varepsilon_{T}T_{r}}\!\left[\!(d_{T_{1}}T_{1}\,+\,d_{T_{8}}T_{8}\,+\,\varepsilon_{T}d_{T_{r}}T_{r})\,+\,\varepsilon_{T}\delta_{TI_{6}}\!\nabla\! \cdot\! (T_{r}\nabla I_{6})\right]\\ & -\underbrace{\mu_{PA} PA,}_{\textrm{depletion by anti-PD-1}} \end{aligned}}  $$

where *μ*_*PA*_ is the depletion rate of PD-1 by anti-PD-1.

PD-L1 is expressed on the surface of activated CD 4^+^ T cells, activated CD 8^+^ T cells, MDSCs, and tumor cells. We assume that the number of PD-L1 per cell is the same for *T*_1_, *T*_8_ and *M* cells, and denote the ratio between the mass of one PD-L1 protein to the mass of one cell by *ρ*_*L*_. Then

14 $$  L=\rho_{L}(T_{1}+T_{8}+\varepsilon_{M}M+\varepsilon_{C} C),  $$

where *ε*_*C*_ depends on the specific type of tumor.

PD-L1 from T cells or cancer cells combines with PD-1 on the plasma membrane of T cells, thus forming a complex PD-1-PD-L1 (*Q*) on the T cells [2, 3]. Denoting the association and disassociation rates of *Q* by *α*_*PL*_ and *d*_*Q*_, respectively, we can write

$$ P+L \overset{\alpha_{PL}}{\underset{d_{Q}}{\rightleftharpoons}} Q. $$

The half-life of *Q* is less then 1 second (i.e. 1.16×10^−5^ day) [43], so that *d*_*Q*_ is very large. Hence we may approximate the dynamical equation for *Q* by the steady state equation, *α*_*PL*_ *P* *L*=*d*_*Q*_ *Q*, or

15 $$  Q=\sigma PL,  $$

where *σ*=*α*_*PL*_/*d*_*Q*_.

#### Equation for anti-PD-1 (*A*)

We assume that anti-PD-1 is injected intradermally every three days for 30 days (as in mouse experiments [44]), providing a source $\hat A(t)$ of anti-PD-1:

$$\hat A(t)=\left\{\begin{array}{ll} \gamma_{A} & \text{if}\: t\le 30,\\ \gamma_{A}\times \frac{33-t}{3} & \text{if}\: 30<t\le 33,\\ 0 & \text{if}\: t>33. \end{array}\right. $$

where *γ*_*A*_ is the effective level of the drug; although the level of the drug varies between injections, for simplicity we take it to be constant. The drug *A* is depleted in the process of blocking PD-1. Hence,

16 $$\begin{array}{@{}rcl@{}}  {}\frac{\partial A}{\partial t}-\delta_{A} \nabla^{2} A=\hat A(t) -\!\!\!\!\!\!\!\!\!\!\!\!\!\!\underbrace{\mu_{PA} PA}_{\text{depletion through blocking PD-1}}-\underbrace{d_{A}A}_{\text{degradation}}.\\ \end{array} $$

#### Equation for BRAF/MEK inhibitor (*B*)

We assume that the BRAF/MEK inhibitor is injected intradermally every days for 30 days, providing a source $\hat B(t)$ of BRAF/MKEi:

$$\hat B(t)=\left\{\begin{array}{ll} \gamma_{B} & \text{if}\: t\le 30,\\ \gamma_{B}\times \frac{33-t}{3} & \text{if}\: 30<t\le 33,\\ 0 & \text{if}\: t>33. \end{array}\right. $$

Assuming that BRAF/MEKi is absorbed by *C* at a rate $\mu _{BC}C\frac {B}{K_{B}+B}$, we get the following equation for *B*:

17 $$ {}\begin{aligned} \frac{\partial B}{\partial t}-\delta_{B} \nabla^{2} B=&\hat B(t) -\underbrace{\mu_{BC}C\frac{B}{K_{B}+B}}_{\text{absorption by cancer cells}}-\underbrace{d_{B}B}_{\text{degradation}}. \end{aligned}  $$

#### Equation for cells velocity (**u**)

We assume that a part of the tumor consists of extracellular matrix, ECM (approximately, 0.4 g/ cm^3^), cancer cells (approximately, *C*=0.4 g/cm^3^) and MDSCs (approximately, *M*=0.2 g/cm^3^). We assume (in the section of parameter estimation) that the densities of the immune cells *D*, *T*_1_, *T*_8_ and *T*_*r*_ are approximately 4×10^−4^, 2×10^−3^, 1×10^−3^ g/ cm^3^ and 5×10^−4^ g/ cm^3^, respectively, and, for consistency, take the constant in Eq. (1) to be 0.6039. We further assume that all cells have approximately the same volume and surface area, so that the diffusion coefficients of all the cells are the same. Adding Eqs. (2)-(7), we then get

18 $$  0.6039\times \nabla\cdot \mathbf{u}=\sum\limits_{j=2}^{7} \left[\text{RHS \: of\: Eq.\:(j)} \right].  $$

To simplify the computations, we assume that the tumor is spherical and denote its radius by *r*=*R*(*t*). We also assume that all the densities and concentrations are radially symmetric, that is, functions of (*r*,*t*), where 0≤*r*≤*R*(*t*). In particular, **u**=*u*(*r*,*t*)**e**_*r*_, where **e**_*r*_ is the unit radial vector.

#### Equation for free boundary (*R*)

We assume that the free boundary *r*=*R*(*t*) moves with the velocity of cells, so that

19 $$  \frac{dR(t)}{dt}=u(R(t),t).  $$

#### Boundary conditions

We assume that the naive CD 4^+^ T cells and inactive CD 8^+^ T cells that migrated from the lymph nodes into the tumor microenvironment have constant densities $\hat T_{1}$ and $\hat T_{8}$ at the tumor boundary, and that *T*_1_ and *T*_8_ are activated by IL-12 upon entering the tumor. We then have the following flux conditions at the tumor boundary:

20 $$  \begin{aligned} \frac{\partial T_{1}}{\partial r}+\sigma_{T}(I_{12})(T_{1}-\hat T_{1})&=0,\\ \frac{\partial T_{8}}{\partial r}+\sigma_{T}(I_{12})(T_{8}-\hat T_{8})&=0\:\:\text{at}\:\: r=R(t), \end{aligned}  $$

where $\sigma _{T}(I_{12})=\sigma _{0} \frac {I_{12}}{I_{12}+K_{I_{12}}}$.

We impose a no-flux boundary condition for all the remaining variables:

21 $$ {{}\begin{aligned} \text{No-flux for} &\:D,\:T_{r},\: M,\: C,\: I_{12},\: I_{2},\: T_{\beta},\: I_{6},\: I_{10},\: P,\: A,\:\text{and} \:B\: \text{at}\: r=R(t). \end{aligned}}  $$

It is tacitly assumed here that the receptors PD-1 and ligands PD-L1 become active only after the T cells are already inside the tumor.

#### Initial conditions

Later on we shall compare the simulations of the model with experimental results for mice, for 60 days. Accordingly, we take initial values whereby the average density of cancer cells has not yet increased to its steady state. Then, by Eq. (1), the total density of the immune cells is initially above its steady state. We take (in unit of g/ cm^3^):

22 $$  \begin{aligned} D&=2\times 10^{-4}, \: T_{1}=4\times 10^{-3},\: T_{8}=2\times 10^{-3},\\ T_{r}&=3\times 10^{-3}, \: M=0.22, \: C=0.3774. \end{aligned}  $$

Note that the initial conditions for the cells satisfy Eq. (1).

We assume that initially *B*=0 and *A*=0, and take the initial condition for *I*_12_, *I*_2_, *T*_*β*_, *I*_6_, *I*_10_ and *P* to be close to their steady state values, which are computed in the section on parameter estimation. One choice of initial conditions is given as follows (in unit of g/ cm^3^):

$$\begin{array}{@{}rcl@{}}  I_{12}&=&4\times 10^{-10}, \: I_{2}=4.74\times 10^{-11},\: T_{\beta}=2.62\times 10^{-13},\\ I_{6}&=&3.06\!\times \! 10^{-11},\: I_{10}=9.66\times 10^{-11},\:P=8.3\times 10^{-10}. \end{array} $$

However, other choices of these initial conditions do not affect the simulations of the model after a few days.

## Results and discussions

The simulations of the model were performed by Matlab based on the moving mesh method for solving partial differential equations with free boundary [45] (see the section on computational method).

**Fig. 2:**
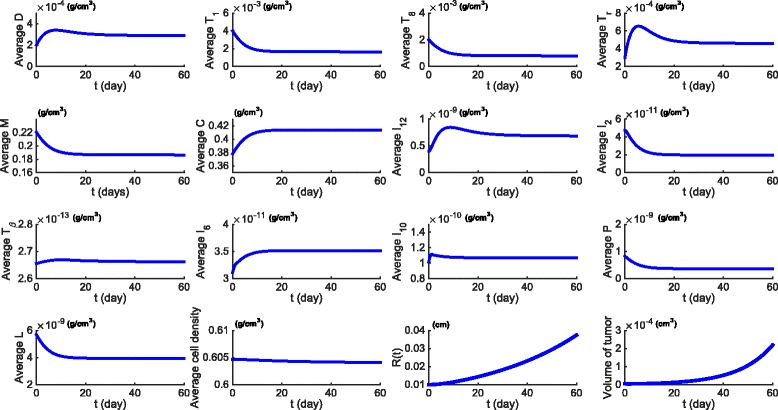
Average densities/concentrations of all the variables in the model in the control case (no drugs). All parameter values are the same as in Tables 2 and 3

Figure 2 is a simulation of the model with no drugs (the control case) for the first 60 days. The average density or concentration of a species is computed as its total mass in the tumor divided by the tumor volume. The simulation shows consistency in the choice of the model parameters. Indeed, as can be quickly checked, the steady states of all the cytokines and cells are approximately equal to the half-saturation values that we assumed in estimating the parameters of the model. Furthermore, the volume of the tumor doubles approximately every 10 days, as was assumed in the choice of the parameter *λ*_0_ (used in estimating some parameters of Eq. (7)). It is interesting to note that the initial increase in TGF- *β* more than compensates for the initial decrease in *P* and *L*, as evident by the initial increase in *T*_*r*_. This initial increase of *T*_*r*_ results in initial decrease in the *T*_1_ and *T*_8_ cells. We also note that the initial increase in cancer cells results in an increase in the *D* cells.

**Table 2:** Summary of parameter values

Notation	Description	Value used	References
*δ*_*D*_	Diffusion coefficient of DCs	8.64×10^−7^ cm^2^ day^−1^	[65]
*δ*_*T*_	Diffusion coefficient of T cells	8.64×10^−7^ cm^2^ day^−1^	[65]
*δ*_*M*_	Diffusion coefficient of MDSCs	8.64×10^−7^ cm^2^ day^−1^	[65]
*δ*_*C*_	Diffusion coefficient of tumor cells	8.64×10^−7^ cm^2^ day^−1^	[65]
$\delta _{I_{12}}$	Diffusion coefficient of IL-12	6.05×10^−2^ cm^2^ day^−1^	Estimated
$\delta _{I_{2}}$	Diffusion coefficient of IL-2	9.58×10^−2^ cm^2^ day^−1^	Estimated
$\delta _{T_{\beta }}$	Diffusion coefficient of TGF- *β*	8.52×10^−2^ cm^2^ day^−1^	Estimated
$\delta _{I_{6}}$	Diffusion coefficient of IL-6	9.03×10^−2^ cm^2^ day^−1^	Estimated
$\delta _{I_{10}}$	Diffusion coefficient of IL-10	9.11×10^−2^ cm^2^ day^−1^	Estimated
*δ*_*A*_	Diffusion coefficient of anti-PD-1	7.85×10^−2^ cm^2^ day^−1^	Estimated
*δ*_*B*_	Diffusion coefficient of BRAF/MEKi	3.16×10^−1^ cm^2^ day^−1^	Estimated
*σ*_0_	Flux rate of *T*_1_ and *T*_8_ cells at the boundary	1 cm^−1^	[65]
*χ*	Chemoattraction coefficient of IL-6	10 cm^5^/g·day	[90, 91]
*λ*_*DC*_	Activation rate of DCs by tumor cells	4 g/cm^3^·day	[65]
$\lambda _{T_{1}I_{12}}$	Activation rate of CD 4^+^ T cells by IL-12	18.64 day^−1^	Estimated
$\lambda _{T_{1}I_{2}}$	Activation rate of CD 4^+^ T cells by IL-2	0.25 day^−1^	[65]
$\lambda _{T_{8}I_{12}}$	Activation rate of CD 8^+^ T cells by IL-12	16.6 day^−1^	Estimated
$\lambda _{T_{8}I_{2}}$	Activation rate of CD 8^+^ T cells by IL-2	0.25 day^−1^	[65]
$\lambda _{T_{r}T_{\beta }}$	Activation rate of Tregs by TGF- *β*	0.415 day^−1^	Estimated
$\lambda _{T_{r}Q}$	Activation rate of Tregs by PD-1-PD-L1	0.083 day^−1^	Estimated
*λ*_*M*_	Activation rate of MDSCs	1.05 day^−1^	[40]
*λ*_*C*_	Growth rate of cancer cells	0.616 day^−1^	Estimated
*λ*_0_	Growth rate of cancer cells uninhibited (by immune cells)	0.069 day^−1^	Estimated
$\lambda _{I_{12}D}$	Production rate of IL-12 by DCs	2.76×10^−6^ day^−1^	Estimated
$\lambda _{I_{12}B}$	Promotion of IL-12 production by BRAF/MEKi	1	Estimated
$\lambda _{I_{2}T_{1}}$	Production rate of IL-2 by CD 4^+^ T cells	2.82×10^−8^ day^−1^	Estimated
$\lambda _{T_{\beta } C}$	Production rate of TGF- *β* by cancer cells	2.18×10^−10^ day^−1^	Estimated
$\lambda _{T_{\beta } T_{r}}$	Production rate of TGF- *β* by Tregs	5.57×10^−9^ day^−1^	[75]
$\lambda _{T_{\beta } M}$	Production rate of TGF- *β* by MDSCs	2.18×10^−10^ day^−1^	Estimated
$\lambda _{I_{6} C}$	Production rate of IL-6 by cancer cells	3.54×10^−10^ day^−1^	Estimated
$\lambda _{I_{10} C}$	Production rate of IL-10 by cancer cells	9.10×10^−10^ day^−1^	Estimated
$\lambda _{I_{10} M}$	Production rate of IL-10 by MDSCs	1.82×10^−9^ day^−1^	Estimated
*η*_1_	Killing rate of tumor cells by CD 4^+^ T cells	11.5 day^−1^·cm^3^/g	Estimated
*η*_8_	Killing rate of tumor cells by CD 8^+^ T cells	46 day^−1^·cm^3^/g	Estimated
*μ*_*PA*_	Blocking rate of PD-1 by anti-PD-1	6.04×10^6^ cm^3^/g·day	Estimated
*μ*_*BC*_	Absorbtion rate of BRAF/MEKi by cancer cells	6.17×10^−10^ day^−1^	Estimated
*ρ*_*P*_	Expression of PD-1 in T cells	2.49×10^−7^	Estimated
*ρ*_*L*_	Expression of PD-L1 in T cells	5.22×10^−7^	Estimated
*ε*_*C*_	Expression of PD-L1 in tumor cells	0.01	[84]
*ε*_*M*_	Expression of PD-L1 in MDSCs	0.005	Estimated
*d*_*D*_	Death rate of DCs	0.1 day^−1^	[65]
$d_{T_{1}}$	Death rate of CD 4^+^ T cells	0.197 day^−1^	[65]
$d_{T_{8}}$	Death rate of CD 8^+^ T cells	0.18 day^−1^	[65]
$d_{T_{r}}$	Death rate of Tregs	0.2 day^−1^	[75]
*d*_*M*_	Death rate of MDSCs	0.03 day^−1^	[40]
*d*_*C*_	Death rate of tumor cells	0.17 day^−1^	[65]
$d_{I_{12}}$	Degradation rate of IL-12	1.38 day^−1^	[65]
$d_{I_{2}}$	Degradation rate of IL-2	2.376 day^−1^	[65]
$d_{T_{\beta }}$	Degradation rate of TGF- *β*	499.066 day^−1^	Estimated
$d_{I_{6}}$	Degradation rate of IL-6	4.16 day^−1^	Estimated
$d_{I_{10}}$	Degradation rate of IL-10	8.32 day^−1^	Estimated
*d*_*A*_	Degradation rate of anti-PD-1	0.046 day^−1^	[87]
*d*_*B*_	Degradation rate of BRAF/MEKi	1.66day^−1^	Estimated
*D*_0_	Density of inactive DCs	2×10^−5^ g/cm^3^	[65]
*T*_10_	Density of naive CD 4^+^ T cells in tumor	4×10^−4^ g/cm^3^	Estimated
*T*_80_	Density of naive CD 8^+^ T cells in tumor	2×10^−4^ g/cm^3^	Estimated
*C*_*M*_	Carrying capacity of cancer cells	0.8 g/cm^3^	[65]
$\hat T_{1}$	Density of CD 4^+^ T cells from lymph node	4×10^−3^ g/cm^3^	Estimated
$\hat T_{8}$	Density of CD 8^+^ T cells from lymph node	2×10^−3^ g/cm^3^	Estimated

**Table 3:** Summary of parameter values

Notation	Description	Value used	References
$K_{T_{1}}$	Half-saturation of CD 4^+^ T cells	2×10^−3^ g/cm^3^	Estimated
$K_{T_{8}}$	Half-saturation of CD 8^+^ T cells	1×10^−3^ g/cm^3^	Estimated
$K_{T_{r}}$	Half-saturation of Tregs	5×10^−4^ g/cm^3^	[65]
*K*_*C*_	Half-saturation of tumor cells	0.4 g/cm^3^	[65]
$K_{I_{12}}$	Half-saturation of IL-12	8×10^−10^ g/cm^3^	Estimated
$K_{I_{2}}$	Half-saturation of IL-2	2.37×10^−11^ g/cm^3^	[65]
$K_{T_{\beta }}$	Half-saturation of TGF- *β*	2.68×10^−13^ g/cm^3^	Estimated
$K_{I_{6}}$	Half-saturation of IL-6	3.4×10^−11^ g/cm^3^	Estimated
$K_{I_{10}}$	Half-saturation of IL-10	8.75×10^−11^ g/cm^3^	Estimated
$K^{\prime }_{Q}$	Half-saturation of PD-1-PD-L1	3.54×10^−18^ g^2^/cm^6^	Estimated
*K*_*B*_	Half-saturation of BRAF/MEKi	6.69×10^−10^ g/cm^3^	Estimated
$K^{\prime }_{TQ}$	Inhibition of function of T cells by PD-1-PD-L1	1.77×10^−18^ g^2^/cm^6^	Estimated
*K*_*CB*_	Inhibition of proliferation of cancer cells by BRAF/MEKi	3.06×10^−9^ g/cm^3^	Estimated

**Fig. 3:**
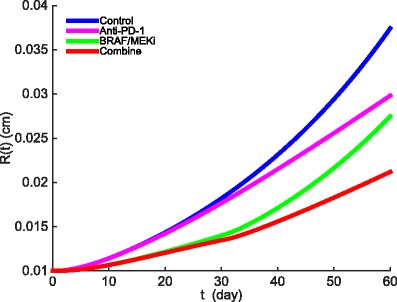
The growth of tumor radius *R*(*t*) during the administration of anti-PD-1 drug and BRAF/MEK inhibitors. Anti-PD-1 is administered at rate *γ*_*A*_=0.3×10^−9^ g/cm^3^·day and BRAF/MEK inhibitor is administered at rate *γ*_*B*_=0.5×10^−9^ g/cm^3^·day. All other parameter values are the same as in Tables 2 and 3

Figure 3 shows the growth of the tumor radius during 60 days when drug is administered. With no drugs, the radius increases from 0.01 cm to 0.037 cm. Treatment with BRAF/MEK inhibitor alone decreased the radius growth more than anti-PD-1 alone, and the combined therapy did better than anti-PD-1 alone. These results agree with mouse experiments reported in [44].

**Fig. 4:**
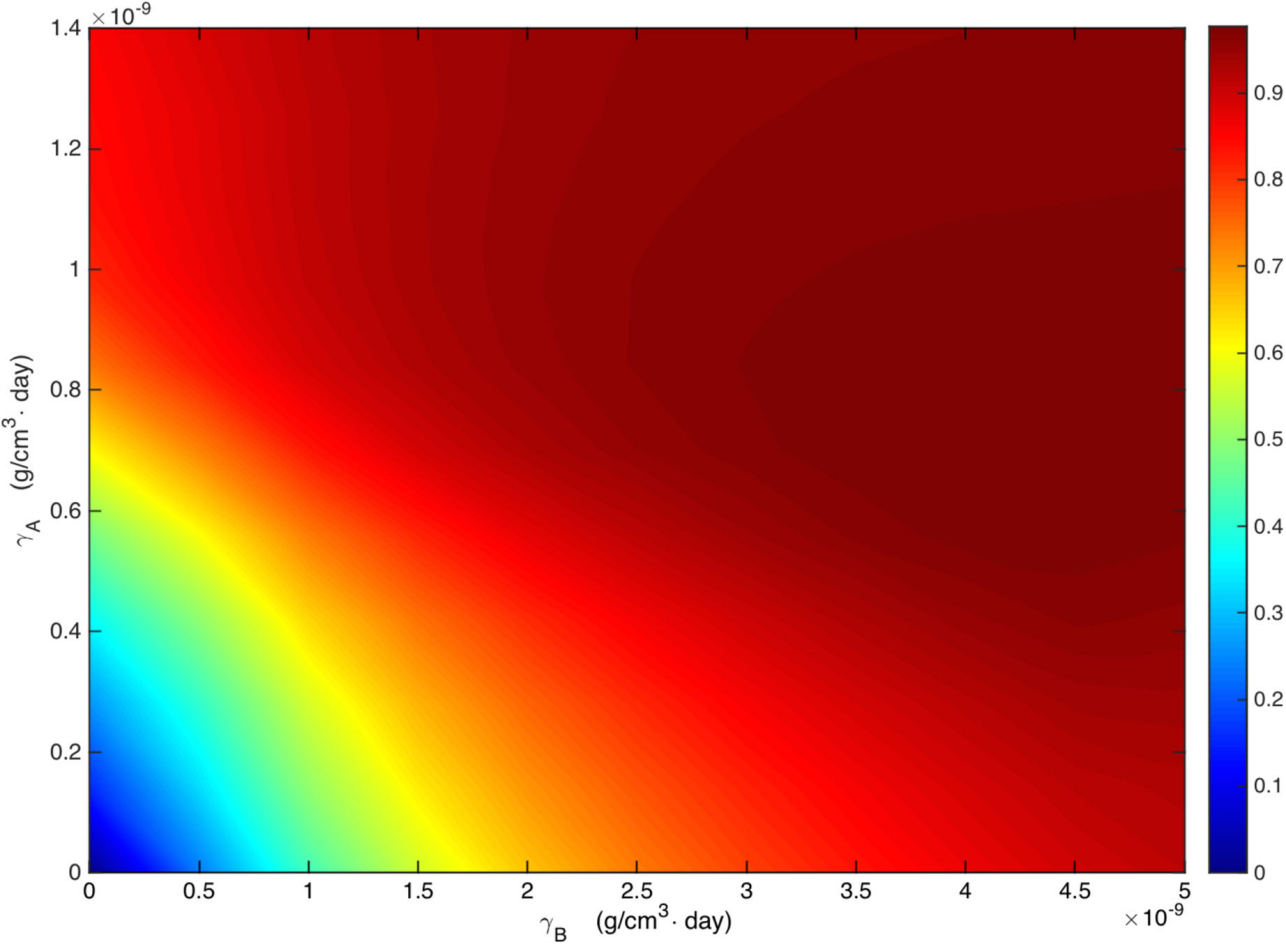
Drug efficacy map. The color column shows the efficacy *E*(*γ*_*B*_,*γ*_*A*_) when *γ*_*B*_ varies between 0−5×10^−9^ g/cm^3^·day and *γ*_*A*_ varies between 0−1.4×10^−9^ g/cm^3^·day. All other parameter values are the same as in Tables 2 and 3

We next consider combination therapy for a range of values of BRAF/MEK inhibitor and anti-PD-1. We define the efficacy of a combination therapy, with (*γ*_*B*_,*γ*_*A*_), by the formula:

$$E(\gamma_{B},\gamma_{A})=\frac{R_{60}(0,0)-R_{60}(\gamma_{B},\gamma_{A})}{R_{60}(0,0)}, $$

where the tumor radius *R*_60_=*R*_60_(*γ*_*B*_,*γ*_*A*_) is computed at day 60; *R*_60_(0,0) is the radius at day 60 in the control case (no drugs). The efficacy is a positive number, and its value lies between 0 and 1 (or between 0 and 100%). Figure 4 is the efficacy map of the combined therapy, with *γ*_*B*_ in the range of 0−5×10^−9^ g/cm^3^·day and *γ*_*A*_ in the range of 0−1.4×10^−9^ g/cm^3^·day. The color column shows the efficacy for any pair of (*γ*_*B*_,*γ*_*A*_); the maximum efficacy is 0.97 (97%).

**Fig. 5:**
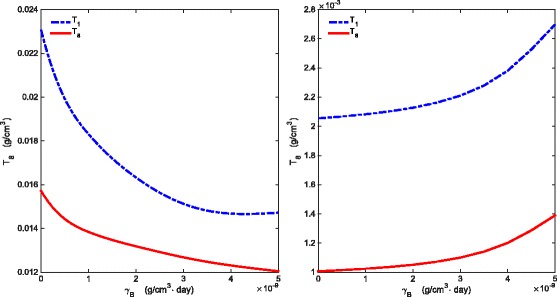
Average densities of *T*_1_ and *T*_8_. **a** Average densities of *T*_1_ and *T*_8_ decrease as *γ*_*B*_ increases for fixed *γ*_*A*_=1.26×10^−9^ g/cm^3^·day; **b**. Average densities of *T*_1_ and *T*_8_ increase as *γ*_*B*_ increases for fixed *γ*_*A*_=0.14×10^−9^ g/cm^3^·day. Here, *γ*_*B*_ varies between 0−5×10^−9^ g/cm^3^·day and all other parameter values are the same as in Tables 2 and 3

**Fig. 6:**
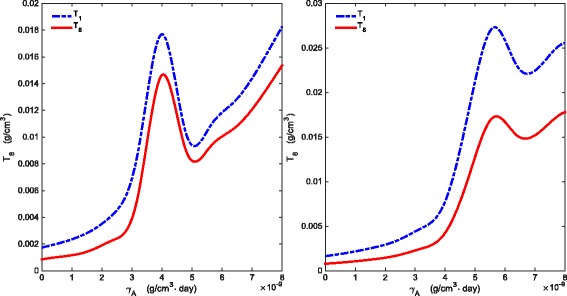
Average densities of *T*_1_ and *T*_8_. **a** There is a *γ*_*A*_-interval where average densities of *T*_1_ and *T*_8_ are decreasing as *γ*_*A*_ increases for fixed *γ*_*B*_=3×10^−9^ g/cm^3^·day. **b** The *γ*_*A*_-interval where average *T*_1_ and *T*_8_ are decreasing may shrink as *γ*_*B*_ is taken to be smaller, e.g. *γ*_*B*_=0.1×10^−9^ g/cm^3^·day. Here, *γ*_*A*_ varies between *n*.4×10^−9^ g/cm^3^·day and all other parameter values are the same as in Tables 2 and 3

 As the number of cancer cells increases, the tumor radius increases. Hence, if *T*_1_ and *T*_8_ were monotone increasing functions of *γ*_*A*_(or of *γ*_*B*_), then we should see that *R*_60_(*γ*_*B*_,*γ*_*A*_) is a decreasing function of *γ*_*A*_(or of *γ*_*B*_), and *E*(*γ*_*B*_,*γ*_*A*_) would then also be an increasing function of *γ*_*A*_(or of *γ*_*B*_). But Fig. 4 shows that this is not generally the case; indeed there are small oscillations in “northeast” corner of the figure. This means that the functions *T*_1_ and *T*_8_ cannot be monotone increasing with respect to *γ*_*B*_ for fixed *γ*_*A*_>0.5×10^−9^ g/cm^3^·day, and also cannot be monotone increasing in *γ*_*A*_ for fixed *γ*_*B*_>1.5×10^−9^ g/cm^3^·day. Indeed, for example, Fig. 5a shows that the average densities of *T*_1_ and *T*_8_ are decreasing functions of *γ*_*B*_, for fixed *γ*_*A*_=1.26×10^−9^ g/cm^3^·day; however, for smaller values of *γ*_*A*_, *T*_1_ and *T*_8_ may become monotone increasing, as seen, for example, in Fig. 5b with *γ*_*A*_=0.14×10^−9^ g/cm^3^·day. Similarly, Fig. 6a shows that, for fixed *γ*_*B*_=3×10^−9^ g/cm^3^·day, there is a *γ*_*A*_-interval where *T*_1_ and *T*_8_ are decreasing as *γ*_*A*_ increases. The *γ*_*A*_-interval where *T*_1_ and *T*_8_ are decreasing may shrink as we take a smaller fixed *γ*_*B*_, as seen, for example, in Fig. 6b with *γ*_*B*_=0.1×10^−9^ g/cm^3^·day.

**Fig. 7:**
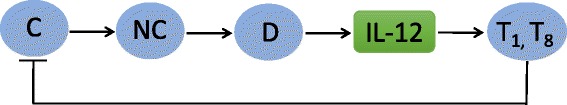
Antagonistic pathway between *C* and (*T*_1_,*T*_8_)

A possible explanation for Fig. 5a is based on the antagonistic pathway shown in Fig. 7. When *γ*_*B*_ increases, the population of cancer cells decreases, and then, by Eqs. (2)-(4) and (8), so does the signal to activate T cells by dendritic cells-derived IL-12 (since the number of activated dendritic cells decrease with decreased cancer cell density) and thus the densities of *T*_1_ and *T*_8_ decrease. As for Fig. 6a, when *γ*_*A*_ begins to increase, *T*_1_ and *T*_8_ also begin to increase, which results in a decrease of cancer cells. Then, as explained in the case of Fig. 5a, this leads to a decrease in dendritic cells-derived IL-12 and, hence, the density of activated *T*_1_ and *T*_8_ cells will begin to decrease as *γ*_*A*_ continues to increase for a while.

If we inject IL-12 directly into tumor (as an additional drug), the influence of dendritic cells-secreted IL-12 diminishes, and the antagonism between BRAF/MEKi and anti-PD-1 also diminishes and it disappears already at very small amount of injection, e.g., an injection of order of magnitude 10^−14^ gcm^3^·day.

## Sensitivity analysis

**Fig. 8:**
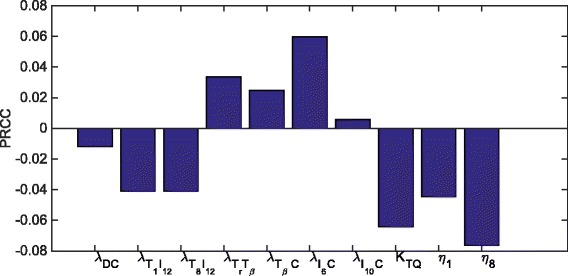
Statistically significant PRCC values (*p*-value <0.01) for *R*(*t*) at day 60

We performed sensitivity analysis, with respect to the rumor radius *R* at day 60 in the control case, with respect to some of the production parameters of the system (2)-(16), namely, *λ*_*DC*_, $\lambda _{T_{1}I_{12}}$, $\lambda _{T_{8}I_{12}}$, $\lambda _{T_{r}T_{\beta }}$, $\lambda _{T_{\beta } C}$, $\lambda _{I_{6}C}$, $\lambda _{I_{10}C}$,and the parameters *K*_*TQ*_, *η*_1_ and *η*_8_ which play important role in the dynamics of *C*. Following the method of [46], we performed Latin hypercube sampling and generated 1000 samples to calculate the partial rank correlation coefficients (PRCC) and the *p*-values with respect to the tumor radius at day 60. In sampling all the parameters, we took the range of each from 1/2 to twice its values in Tables 2 and 3. The results are shown in Fig. 8.

We see that the production/activation rates that promote effector T cells, namely, *λ*_*DC*_, $\lambda _{T_{1}I_{12}}$ and $\lambda _{T_{8}I_{12}}$, are negatively correlated to the tumor radius, while the production/activation rates of the effector T cell-suppressors, such as $\lambda _{T_{r}T_{\beta }}$, $\lambda _{I_{10}C}$, $\lambda _{T_{\beta } C}$ and $\lambda _{I_{6}C}$, are positively correlated to the tumor radius. The killing rate of effector T cells, *η*_1_ and *η*_8_ are negatively correlated to the tumor radius, and the correlation with *η*_8_ is higher than with *η*_1_.

## Conclusion

BRAF mutation occurs in up to 66% of human malignant melanomas and for this reason BRAF has been one of the primary targets in melanoma therapy. Treatment with BRAF inhibitors (such as vemurafenib or dabrafenib) encounters MAPK-driven resistance, but combining it with MEK inhibitor (e.g. trametinib) significantly reduces this resistance as well as toxicity. While the response to the combined BRAF/MEK inhibitor is significant, it is short lived. On the other hand, PD-1 antibody (nivolumab) has lower response rate but a far greater durability. It was therefore suggested that BRAF/MEK inhibitor should positively correlate with anti-PD-1.

In the present paper we developed a mathematical model to test this hypothesis, *in silico*, by computing the efficacy of the combined therapy. The model is represented by a system of partial differential equations within the tumor tissue. The model includes immune cells (Th1 and CD 8^+^ T cells, Tregs, MDSCs and dendritic cells), cytokines (IL-12, IL-2, IL-6, IL-10 and TGF- *β*), and PD-1, PD-L1 and the complex PD-1-PD-L1. We simulated the model with combination of drugs, BRAF/MEK inhibitor at the ‘level’ *γ*_*B*_ and PD-1 antibody at the ‘level’ *γ*_*A*_, and computed the tumor radius *R*_60_=*R*_60_(*γ*_*A*_,*γ*_*B*_) at day 60, and the efficacy $E(\gamma _{B},\gamma _{A})=\frac {R_{60}(0,0)-R_{60}(\gamma _{B},\gamma _{A})}{R_{60}(0,0)}$; the efficacy is an expression that quantifies the reduction in tumor size compared to the control case (no drugs).

The efficacy map in Fig. 4 shows that for low levels of *γ*_*B*_ and *γ*_*A*_, the two drugs are positively correlated, in the sense that tumor volume decreases as each of the drugs is increased. However, in the ‘northeast’ corner of Fig. 4 we see that for higher levels of *γ*_*B*_ and *γ*_*A*_ there are zones where the drugs are antagonistic in the sense that when *γ*_*B*_ and *γ*_*A*_ in these zones are increased, the efficacy actually decreases. The antagonism between the combined drugs can be explained by the pathway shown in Fig. 7. An increase in the number of effector T cells (Th1 and CD 8^+^) results in decrease in cancer cells and necrotic cancer cells, hence in decreased signals to activate dendritic cells. This results in a decrease in IL-12 production by dendritic cells, and hence in a decrease in effector T cells.

The parameter $\lambda _{I_{12}B}$ may be viewed as the immune system response to BRAF/MEK inhibitor. When this parameter is increased, the antagonism in the combined therapy is reduced, but it does not completely disappear (not shown here).

The mathematical model presented in this paper has several limitations:

(i) In order to focus on the combined therapy of a BRAF/MEK inhibitor and an anti-PD-1 drug, we did not include in the model the effect of angiogenesis, thus assuming that the tumor is avascular. We tacitly assumed that the effect of this omission is not significant in comparing the results of therapy to no therapy.
(ii) We assumed that the densities of immature, or naive, immune cells remain constant throughout the progression of the cancer and that density of debris of dead cells is constant.
(iii) We assumed that the process of necrosis is fast, and that the density of cancer cells undergoing necrosis is at steady state.
(iv) In estimating parameters we made a steady state assumption in some of the differential equations.
(v) We did not make any direct connection between drugs administered to the patient, and their ‘effective strengths’ *γ*_*B*_ and *γ*_*A*_, as they appear in the differential equations, since these data are not available.

A general study of synergistic and antagonistic networks in drug combinations appeared in [47]. Clinical records on combination therapy show that the number of drugs that are synergistic far exceeds the number of drugs that are antagonistic [48].

In our model, the combination (*γ*_*B*_,*γ*_*A*_) is antagonistic when the drugs are administered in high doses, but not in low doses. For this reason it will be important to identify more carefully the zones of antagonism, by animal experiments or by early clinical trials, in order to avoid those zones in more advanced clinical trials.

## Appendix

### Parameter estimation

#### Half-saturation

In an expression of the form $Y\frac {X}{K_{X}+X}$ where *Y* is activated by *X*, the half-saturation parameter *K*_*X*_ is taken to be the approximate steady state concentration of species *X*.

#### Diffusion coefficients

By [49], we have the following relation for estimating the diffusion coefficients of a protein *p*:

$$\delta_{p}=\frac{M^{1/3}_{V}}{M^{1/3}_{p}}\delta_{V}, $$

where *M*_*V*_ and *δ*_*V*_ are respectively the molecular weight and diffusion coefficient of VEGF, *M*_*p*_ is the molecular weight of *p*, and *M*_*V*_=24kDa [50] and *δ*_*V*_=8.64×10^−2^ cm^2^ day^−1^ [51]. Since, $M_{I_{2}}=17.6$kDa [52], $M_{I_{12}}=70$kDa [53], $M_{T_{\beta }}=25$kDa [54], $M_{I_{6}}=21$kDa [55, 56], $M_{I_{10}}=20.5$kDa [57], *M*_*A*_=32kDa [58] and *M*_*B*_=489.93Da [59], we get $\delta _{I_{2}}=9.58\times 10^{-2}\ \text {cm}^{2}\:\text {day}^{-1}$, $\delta _{I_{12}} =6.05\times 10^{-2}\ \text {cm}^{2}\:\text {day}^{-1}$, $\delta _{T_{\beta }}=8.52\times 10^{-2}\ \text {cm}^{2}\:\text {day}^{-1}$, $\delta _{I_{6}}=9.03\times 10^{-2}\ \text {cm}^{2}\:\text {day}^{-1}$, $\delta _{I_{10}}=9.11\times 10^{-2}\ \text {cm}^{2}\:\text {day}^{-1}$, *δ*_*A*_=7.85×10^−2^ cm^2^ day^−1^ and *δ*_*B*_=3.16×10^−1^ cm^2^ day^−1^.

#### Equation (2)

The number of DCs in various organs (heart, kidney, pancreas and liver) in mouse varies from 1.1×10^6^ cells/ cm^3^ to 6.6×10^6^ cells/ cm^3^ [60]. In the dermal tissue, the number of DCs is larger (600-1500 cells/ mm^2^) [61, 62], but we do not specify where the melanoma cancer is located; it may be at its initial dermal tissue or in another organ where it metastasized. Mature DCs are approximately 10 to 15 *μ*m in diameter [63]. Accordingly, we estimate the steady state of DCs to be *K*_*D*_=4×10^−4^ g/cm^3^. We assume that there are always immature dendritic cells, some coming from the blood as tumor infiltrating dendritic cells (TID) [20, 21, 64]. We also assume that the density of immature DCs to be smaller than the density of active DCs, and take $D_{0}=\frac {1}{20}K_{D}=2\times 10^{-5}\ \mathrm {g}/\text {cm}^{3}$. From the steady statenof Eq. (2), we get *λ*_*DC*_=2*d*_*D*_ *D*/*D*_0_=4/day, since *d*_*D*_=0.1/day [65]. We take *K*_*C*_=0.4 g/cm^3^.

#### Equation (3)

The number of lymphocytes is approximately twice the number of DCs [60]. T cells are approximately 14 to 20 *μ*m in diameter. Assuming that the number of Th1 cells is 1/4 the number of lymphocytes, we estimate steady state density of Th1 cells to be $K_{T_{1}}=2\times 10^{-3}\ \mathrm {g}/\text {cm}^{3}$. We assume that the density of naive CD 4^+^ T cells to be less than the density of Th1, and take $T_{10}=\frac {1}{5}K_{T}=4\times 10^{-4}\ \mathrm {g}/\text {cm}^{3}$. As in [65], we choose $K_{TT_{r}}$ to be half-saturation of *T*_*r*_, that is, $K_{TT_{r}}=5\times 10^{-4}\ \mathrm {g}/\text {cm}^{3}$, and as in [66], we choose $K_{TI_{10}}$ to be half-saturation of *I*_10_, namely, $K_{TI_{10}}=2\times 10^{-7}\ \mathrm {g}/\text {cm}^{3}$. We assume that in steady state, *Q*/*K*_*TQ*_=2 (the value of *K*_*TQ*_ is derived in the estimates of Eqs. (13)-(15)). From the steady state of Eq. (3), we get

$$\left(\lambda_{T_{1}I_{12}}T_{10}\cdot\frac{1}{2}\cdot\frac{1}{2}\cdot\frac{1}{2}+\lambda_{T_{1}I_{2}}T_{1}\cdot\frac{1}{2}\right)\cdot\frac{1}{3}-d_{T_{1}}T_{1}=0, $$

where $\lambda _{T_{1}I_{2}}=0.25$/day [65], $d_{T_{1}}=0.197$/day [65], *T*_10_=4×10^−4^ g/cm^3^ and $T_{1}=K_{T_{1}}=2\times 10^{-3}\ \mathrm {g}/\text {cm}^{3}$. Hence $\lambda _{T_{1}I_{12}}=18.64/\text {day}$.

#### Equation (4)

The CD4/CD8 ratio in the blood is 2:1. We assume a similar ratio in tissue, and take $T_{80}=\frac {1}{2}T_{10}=2\times 10^{-4}\ \mathrm {g}/\text {cm}^{3}$. We also take steady state of *T*_8_ to be the half of steady state of *T*_1_, i.e., $K_{T_{8}}=\frac {1}{2}K_{T_{1}}=1\times 10^{-3}\ \mathrm {g}/\text {cm}^{3}$. From the steady state of Eq. (4), we have

$$\left(\lambda_{T_{8}I_{12}}T_{80}\cdot\frac{1}{2}\cdot\frac{1}{2}\cdot\frac{1}{2}+\lambda_{T_{1}I_{2}}T_{8}\cdot\frac{1}{2}\right)\cdot\frac{1}{3}-d_{T_{8}}T_{8}=0 $$

where $\lambda _{T_{8}I_{2}}=0.25$/day [65], $d_{T_{8}}=0.18$/day [65], *T*_80_=2×10^−4^ g/cm^3^, $T_{8}=K_{T_{8}}=1\times 10^{-3}\ \mathrm {g}/\text {cm}^{3}$. Hence $\lambda _{T_{8}I_{12}}=16.6/\text {day}$.

#### Equation (5)

We assume that TGF- *β* activates Tregs more than PD-1-PD-L1 does, and take $\lambda _{T_{r}T_{\beta }}=5\lambda _{T_{r}Q}$. From the steady state of Eq. (5), we get, $(\lambda _{T_{r}T_{\beta }}\cdot \frac {1}{2}+\lambda _{T_{r}Q}\cdot \frac {1}{2})T_{10}-d_{T_{r}}T_{r}=0$, where *T*_10_=1×10^−3^ g/cm^3^, $T_{r}=K_{T_{r}}=5\times 10^{-4}\ \mathrm {g}/\text {cm}^{3}$ [65], and $d_{T_{r}}=0.2$/day [65]. Hence $\lambda _{T_{r}Q}=0.083/\text {day}$ and $\lambda _{T_{r}T_{\beta }}=0.415$/day.

#### Equation (6)

The density of tumor-associated macrophages in melanoma can be up to 30% of the tumor tissue density [67]; we take MDSC density to be 20% of the tumor tissue density, so that *M*=0.2 g/cm^3^ in steady state. From the steady state of Eq. (6), we get, $\frac {1}{2}\lambda _{M}(M_{0}-M)=d_{M}M$, where *d*_*M*_=0.015/day [40], *λ*_*M*_=20/19=1.05 [40], and *M*=*K*_*M*_=0.2 g/cm^3^. Hence, *M*_0_=0.21 g/cm^3^.

#### Equation (7)

We take *d*_*C*_=0.17 day^−1^ and *C*_*M*_=0.8 g/cm^3^ [65]. In the control case (no anti-tumor drugs), the tumor grows according to

23 $$  \frac{dC}{dt}=\lambda_{C} C\left(1-\frac{C}{C_{M}}\right)-(\eta_{1}T_{1}+\eta_{8}T_{8})C-d_{C}C.  $$

Mouse experiments show that tumor volume doubles within 5 -15 days [44, 68–70]. Assuming a linear growth

$$\frac{dC}{dt}=\lambda_{0} C, \: \: \text{where}\: \lambda_{0}>0, $$

during the volume doubling time in the control case, we conclude from Eq. (23) that

24 $$  \lambda_{C} C\left(1-\frac{C}{C_{M}}\right)-(\eta_{1}T_{1}+\eta_{8}T_{8})C-d_{C}C=\lambda_{0} C.  $$

where $\lambda _{0}\in \left (\frac {\text {ln}2}{15},\frac {\text {ln}2}{5}\right)$. We assume that without immune responses and BRAF/MEK inhibitor,

$$\frac{dC}{dt}=2\lambda_{0} C, $$

so that

25 $$  \lambda_{C} C\left(1-\frac{C}{C_{M}}\right)-d_{C}C=2\lambda_{0} C.  $$

We further assume that with immune response and BRAF/MEK inhibitor, the density of cancer cell still grows,

$$\frac{dC}{dt}=\frac{1}{5}\lambda_{0} C, $$

so that

26 $$  {}\lambda_{C} C\left(\!\!1\,-\,\frac{C}{C_{M}}\!\!\right)\cdot\frac{1}{1\,+\,B/K_{CB}}-(\eta_{1}T_{1}+\eta_{8}T_{8})C-d_{C}C=\frac{1}{5}\lambda_{0} C.  $$

We take *λ*_0_=0.069/day, and assume that in steady state, *C* is approximately 0.4 g/cm^3^, so that from Eq. (25) we get $\frac {1}{2}\lambda _{C}-d_{C}=2\lambda _{0}$, or *λ*_*C*_=2(2*λ*_0_+*d*_*C*_)=0.616/day. By comparing Eq. (24) to Eq. (25), we see that *η*_1_ *T*_1_+*η*_8_ *T*_8_=*λ*_0_. Noting that *T*_8_ cells kill cancer cells more effectively than *T*_1_ cells, we take *η*_8_=4*η*_1_, so that (with $T_{1}=K_{T_{1}}=2\times 10^{-3}\ \mathrm {g}/\text {cm}^{3}$ and $T_{8}=K_{T_{8}}=1\times 10^{-3}\ \mathrm {g}/\text {cm}^{3}$) $\eta _{1}=\frac {\lambda _{0}}{T_{1}+4T_{8}}=11.5\ \text {cm}^{3}/\mathrm {g}\cdot \text {day}$ and *η*_8_=46 cm^3^/g·day. From Eq. (26), we have $\frac {1}{2}\lambda _{C}\cdot \frac {1}{1+B/K_{CB}}-(\eta _{1}T_{1}+\eta _{8}T_{8})-d_{C}=\frac {1}{5}\lambda _{0}$. Since *λ*_*C*_=2(2*λ*_0_+*d*_*C*_) and *η*_1_ *T*_1_+*η*_8_ *T*_8_=*λ*_0_, we get $(2\lambda _{0} +d_{C})\cdot \frac {1}{1+B/K_{CB}}-\lambda _{0}-d_{C}=\frac {1}{5}\lambda _{0}$, so that (with *B*=*K*_*B*_=6.69×10^−10^ g/cm^3^) $K_{CB}=B\frac {5d_{C}+6\lambda _{0} }{4\lambda _{0}}=3.06\times 10^{-9}\ \mathrm {g}/\text {cm}^{3}$.

#### Equation (8)

The serum level of IL-12 in melanoma patients varies from 7.5×10^−11^−9.6×10^−11^ g/cm^3^ [71, 72]. We assume that the IL-12 level in tissue is higher, and take $I_{12}=K_{I_{12}}=8\times 10^{-10}\ \mathrm {g}/\text {cm}^{3}$. In the control case (no drugs), from the steady state of Eq. (8), we get $\lambda _{I_{12}D}D-d_{I_{12}}I_{12}=0$, where $d_{I_{12}}=1.38$/day [65] and *D*=*K*_*D*_=4×10^−4^ g/cm^3^. Hence, $\lambda _{I_{12}D}=2.76\times 10^{-6}$/day. In the simulations we take $\lambda _{I_{12}B}=1$, but simulations do not change qualitatively with smaller or larger values of $\lambda _{I_{12}B}$.

#### Equation (9)

From the steady state of Eq. (9), we get $\lambda _{I_{2}T_{1}}T_{1}-d_{I_{2}}I_{2}=0$, where $d_{I_{2}}=2.376$/day [65] and $I_{2}=K_{I_{2}}=2.37\times 10^{-11}\ \mathrm {g}/\text {cm}^{3}$ [65], and $T_{1}=K_{T_{1}}=2\times 10^{-3}\ \mathrm {g}/\text {cm}^{3}$. Hence, $\lambda _{I_{2}T_{1}}=2.82\times 10^{-8}$/day.

#### Equation (10)

The half-life of TGF- *β* is about 2 min [73], that is, *t*_1/2_=0.0014 day, so that $d_{T_{\beta }}=\text {ln}2/t_{1/2}=499.07\ \text {day}^{-1}$. The concentration of serum TGF- *β* in melanoma is 2.68×10^−14^ g/cm^3^ [74]. We assume that the concentration of TGF- *β* in tissue is higher than in serum, and take *T*_*β*_=2.68×10^−13^ g/cm^3^. By [75], $\lambda _{T_{\beta } T_{r}}=5.57\times 10^{-9}$/day. According to [27, 42], melanoma cells secrete more TGF- *β* than MDSC, and we assume that $\lambda _{T_{\beta } C}C=2\lambda _{T_{\beta } M}$M. Hence, from the steady state of Eq. (10) we have, $\lambda _{T_{\beta } C}C+\lambda _{T_{\beta } M}M+\lambda _{T_{\beta } T_{r}}T_{r}=d_{T_{\beta }}T_{\beta }$, or $3\lambda _{T_{\beta } M}M+\lambda _{T_{\beta } T_{r}}T_{r}=d_{T_{\beta }}T_{\beta }$. Thus $\lambda _{T_{\beta } M}=(d_{T_{\beta }}T_{\beta }-\lambda _{T_{\beta } T_{r}}T_{r})/ (3M)=2.18\times 10^{-10}$/day, and $\lambda _{T_{\beta } C}=2\lambda _{T_{\beta } M}M/C= 2.18\times 10^{-10}$/day.

#### Equation (11)

The half-life of IL-6 is less than 6 hours [76], and we take it to be 4 hours, that is, *t*_1/2_=0.17 day, so that $d_{I_{6}}=\text {ln}2/t_{1/2}=4.16\ \text {day}^{-1}$. The concentration of serum IL-6 in melanoma is 3.4×10^−12^ g/cm^3^ [77]. We assume that the concentration of IL-6 in tissue is higher than in serum, and take *I*_6_=3.4×10^−11^ g/cm^3^. From the steady state of Eq. (11), we get $\lambda _{I_{6}C}=d_{I_{6}}I_{6}/C=3.54\times 10^{-10}$/day.

#### Equation (12)

The half-life of IL-10 ranges from 1.1 to 2.6 hours [78]; we take it to be 2 hours, that is, *t*_1/2_=0.08 day, so that $d_{I_{10}}=8.32\ \text {day}^{-1}$. The concentration of serum IL-10 in melanoma is 8.75×10^−12^ g/cm^3^ [79]. We assume that the concentration of IL-10 in tissue is higher than in serum, and take *I*_10_=8.75×10^−11^ g/cm^3^. In melanoma, the tissue concentrations of IL-10 secreted by tumor cells and by macrophages are similar [80], and, accordingly, we assume that $\lambda _{I_{10}C}C=\lambda _{I_{10}M}M$ in steady state. Hence, from the steady state of Eq. (12) we get, $2\lambda _{I_{10}C}C-d_{I_{1}0}I_{10}=0$, so that $\lambda _{I_{10}C}=d_{I_{10}}I_{10}/2C=9.10\times 10^{-10}$/day, and $\lambda _{I_{10}M}=\lambda _{I_{10}C}C/M=1.82\times 10^{-9}$/day.

#### Equations (13)-(15)

In order to estimate the parameters *K*_*TQ*_ (in Eqs. (3) and (4)) and *K*_*Q*_ (in Eq. (5)), we need to determine the steady state concentrations of *P* and *L* in the control case (no drugs). To do that, we begin by estimating *ρ*_*P*_ and *ρ*_*L*_.

By [81], the mass of one PD-1 is *m*_*P*_=8.3×10^−8^ pg= 8.3×10^−20^ g, and by [1] the mass of one PD-L1 is *m*_*L*_=5.8×10^−8^ pg= 5.8×10^−20^ g. We assume that the mass of one T cell is *m*_*T*_=10^−9^ g. By [82], there are 3000 PD-1 proteins and 9000 PD-L1 proteins on one T cell (*T*_1_ or *T*_8_). Since *ρ*_*P*_ *T* is the density of PD-1 (without anti-PD-1 drug), we get $\rho _{P}=3000\times \frac {m_{P}}{m_{T}}=\frac {3000\times (8.3\times 10^{-20})}{10^{-9}}=2.49\times 10^{-7}$, and $\rho _{L}=9000\times \frac {m_{L}}{m_{T}}=\frac {9000\times (5.8\times 10^{-20})}{10^{-9}}=5.22\times 10^{-7}$.

In order to estimate steady state concentration of *P*, we assume that the average densities of *T*_1_, *T*_8_ and *T*_*r*_ are approximately 2×10^−3^, 1×10^−3^ and 5×10^−4^ g/ cm^3^, respectively. PD-1 is expressed by Tregs at higher or lower level than in *T*_1_ and *T*_8_ cells depending on the type of the cancer [83]; we assume that *ε*_*T*_=0.8. Hence, in steady state,

$$\begin{array}{@{}rcl@{}} P&=&\rho_{P}(T_{1}+T_{8}+\varepsilon_{T} T_{r})\\ &=&\!\!(2.49\!\times\! 10^{-7})\!\times\![2\!\times\! 10^{-3}\,+\,1\!\times\! 10^{-3}\,+\,0.8\times (5\!\times 10^{-4})]\\ &=&8.46\times 10^{-10} \mathrm{g}/\text{cm}^{3}. \end{array} $$

The parameter *ε*_*C*_ in Eq. (14) depends on the type of cancer. We take *ε*_*C*_=0.01 [84]. MDSCs express PD-L1 at lower level than tumor cells [85], and accordingly, we assume that $\varepsilon _{M}M=\frac {1}{4}\varepsilon _{C}C$, so that *ε*_*M*_=*ε*_*C*_ *C*/4*M*=*ε*_*C*_/2=0.005. Then, by Eq. (14), we get

$$\begin{array}{@{}rcl@{}} L&=&\rho_{L}(T_{1}+T_{8}+\varepsilon_{M}M+\varepsilon_{C} C)\\ &=&(5.22\times 10^{-7})\times[3\times 10^{-3}\!+0.005\times 0.2 \,+\,0.01\times 0.4]\\ &=&4.176\times 10^{-9}\mathrm{g}/\text{cm}^{3}. \end{array} $$

In steady state with $P=\bar P$, $L=\bar L$ and $Q=\bar Q$, we have, by Eq. (15), $\bar Q=\sigma \bar P\bar L$. We take $K_{TQ}=\frac {1}{2}\bar Q=\frac {1}{2}\sigma \bar P\bar L$. Hence, $Q/K_{TQ}=PL/(\frac {1}{2}\bar P\bar L)$ and

$$\frac{1}{1+Q/K_{TQ}}=\frac{1}{1+PL/(\frac{1}{2}\bar P\bar L)}=\frac{1}{1+PL/K'_{TQ}}, $$

where $K^{\prime }_{TQ}:=\frac {1}{2}\bar P\bar L=\frac {1}{2}\times (8.46\times 10^{-10})\times (4.176\times 10^{-9})=1.77\times 10^{-18}\ \mathrm {g}^{2}/\text {cm}^{6}$. Similarly, $K_{Q}=\bar Q=\sigma \bar P\bar L$, so that in Eq. (5),

$$\frac{Q}{K_{Q}+Q}=\frac{1}{1+K_{Q}/Q}=\frac{1}{1+\bar P\bar L/PL}=\frac{1}{1+K'_{Q}/PL}. $$

where $K^{\prime }_{Q}:=\bar P\bar L=3.54\times 10^{-18}\ \mathrm {g}^{2}/\text {cm}^{6}$.

#### Equations (16)-(17)

In mice experiments [44, 86] different amounts of drugs were injected, and the amount of BRAF/MEK inhibitor was larger than the amount of anti-PD-1. It is difficult to compare the amounts injected into mice with the actual levels of the drugs which appear in Eqs. (16) and (17), since there is no information available on the PK/PD of the drugs. For the choice of *γ*_*A*_=0.3×10^−9^ g/cm^3^·day and *γ*_*B*_=0.5×10^−9^ g/cm^3^·day, we found that the simulations are in qualitative agreement with experiments reported in [44]. We shall accordingly take *γ*_*A*_ in the range of *n*.4×10^−9^ g/cm^3^·day and *γ*_*B*_ in the range of 0−5×10^−9^ g/cm^3^·day.

By [87], the half-life of anti-PD-1 is 15 days, so that $d_{A}=\frac {\text {ln} 2}{15}=0.046\ \text {day}^{-1}$. We assume that 10% of A is used in blocking PD-1, while the remaining 90% degrades naturally. Hence, *μ*_*PA*_ *P* *A*/10*%*=*d*_*A*_ *A*/90*%*, so that

$${}\mu_{PA}=\frac{d_{A}}{9P}=\frac{0.046}{9\times (8.46\times 10^{-10})}=6.04\times 10^{6}\: \text{cm}^{3}/\mathrm{g}\cdot \text{day}. $$

The half-life of BRAF inhibitor (dabrafenib) is 8 hours [88], and the half-life of MEK inhibitor (trametinib) is 33 h [89]. In the combination of BRAF/MEKi, the proportion of MEKi is smaller than BRAFi [44], and accordingly we take the half-life of BRAF/MEKi to be 10 h, so that $d_{B}=\frac {\text {ln} 2}{10/24}=1.66\ \text {day}^{-1}$. We assume that 10% of B is absorbed by cancer cells, while the remaining 90% degrades naturally, so that $\mu _{BC}C\frac {B}{K_{B}+B}/10\%=d_{B}B/90\%$. From Eq. (17), we get *B*≥*γ*_*B*_/*d*_*B*_, and we assume that

$$B\sim \frac{10}{9}\cdot \frac{\gamma_{B}}{d_{B}}, $$

where *d*_*B*_=1.66/day. We take *γ*_*B*_ to be order of magnitude 10^−9^ g/cm^3^·day in the simulations. Hence, *B*=*K*_*B*_=6.69×10^−10^ g/cm^3^ in steady state, and *μ*_*BC*_=2*d*_*B*_ *B*/9*C*=6.17×10^−10^/day.

Eqs. (20): We assume that $\hat T_{1}$ is larger than $K_{T_{1}}$ and take $\hat T_{1}=4\times 10^{-3}\ \mathrm {g}/\text {cm}^{3}$. Similarly, we also assume that $\hat T_{8}$ is larger than $K_{T_{8}}$ and take $\hat T_{8}=2\times 10^{-3}\ \mathrm {g}/\text {cm}^{3}$.

### Computational method

We employ moving mesh method [45] to numerically solve the free boundary problem for the tumor proliferation model. To illustrate this method, we take Eq. (2) as example and rewrite it as the following form:

27 $$\begin{array}{*{20}l} \frac{\partial D(r,t)}{\partial t}=\delta_{D}\Delta D(r,t)-div(\mathbf{u}D)+ F, \quad  \end{array} $$

where *F* represents the term in the right hand side of Eq. (2). Let $r_{i}^{k}$ and $D_{i}^{k}$ denote numerical approximations of i-th grid point and $D(r_{i}^{k},n\tau)$, respectively, where *τ* is the size of time-step. The discretization of Eq. (27) is derived by the fully implicit finite difference scheme:

28 $$ {{}\begin{aligned} \frac{D_{i}^{k+1}-D_{i}^{k}}{\tau}\,=\,\delta_{D}\left(\!D_{rr}\,+\,\frac{2}{r_{i}^{k}}D_{r}\!\right)\,-\, \left(\!\frac{2}{r_{i}^{k+1}}u_{i}^{k+1}\,+\,u_{r}\!\right)\!D_{i}^{k+1}\,-\,u_{i}^{k+1}D_{r}\,+\,F_{i}^{k+1}\!, \end{aligned}}  $$

where $D_{r}=\frac {h_{-1}^{2}D_{i+1}^{k+1}-h_{1}^{2}D_{i-1}^{k+1}-(h_{1}^{2}-h_{-1}^{2})D_{i}^{k+1}}{h_{1}(h_{-1}^{2}-h_{1}h_{-1})}$, $D_{rr}=2\frac {h_{-1}D_{i+1}^{k+1}-h_{1}D_{i-1}^{k+1}+(h_{1}-h_{-1})D_{i}^{k+1}}{h_{1}(h_{1}h_{-1}-h_{-1}^{2})}$,$u_{r}=\frac {h_{-1}^{2}u_{i+1}^{k+1}-h_{1}^{2}u_{i-1}^{k+1}-(h_{1}^{2}-h_{-1}^{2})u_{i}^{k+1}}{h_{1}(h_{-1}^{2}-h_{1}h_{-1})}$, $h_{-1}=r_{i-1}^{k+1}-r_{i}^{k+1}$ and $h_{1}=r_{i+1}^{k+1}-r_{i}^{k+1}$. The mesh moves by $r_{i}^{k+1}=r_{i}^{k}+u_{i}^{k+1}\tau $, where $u_{i}^{k+1}$ is solved by the velocity equation.
